# Local structure and distortions of mixed methane-carbon dioxide hydrates

**DOI:** 10.1038/s42004-020-00441-7

**Published:** 2021-01-19

**Authors:** Bernadette R. Cladek, S. Michelle Everett, Marshall T. McDonnell, Matthew G. Tucker, David J. Keffer, Claudia J. Rawn

**Affiliations:** 1 Department of Materials Science and Engineering, University of Tennessee, Knoxville, Tennessee 37996-2100 USA; 2 Neutron Scattering Division, Oak Ridge National Laboratory, Oak Ridge, Tennessee 37831-6475 USA; 3 Computer Science and Mathematics Division, Oak Ridge National Laboratory, Oak Ridge, Tennessee 37831-6475 USA

**Keywords:** Carbon capture and storage, Characterization and analytical techniques, Natural gas, Computational methods

## Abstract

A vast source of methane is found in gas hydrate deposits, which form naturally dispersed throughout ocean sediments and arctic permafrost. Methane may be obtained from hydrates by exchange with hydrocarbon byproduct carbon dioxide. It is imperative for the development of safe methane extraction and carbon dioxide sequestration to understand how methane and carbon dioxide co-occupy the same hydrate structure. Pair distribution functions (PDFs) provide atomic-scale structural insight into intermolecular interactions in methane and carbon dioxide hydrates. We present experimental neutron PDFs of methane, carbon dioxide and mixed methane-carbon dioxide hydrates at 10 K analyzed with complementing classical molecular dynamics simulations and Reverse Monte Carlo fitting. Mixed hydrate, which forms during the exchange process, is more locally disordered than methane or carbon dioxide hydrates. The behavior of mixed gas species cannot be interpolated from properties of pure compounds, and PDF measurements provide important understanding of how the guest composition impacts overall order in the hydrate structure.

## Abstract (web summary)

Natural gas can be collected from hydrate deposits by exchange with CO_2_, but the ensuing mixed hydrates and the path to a complete exchange are not fully understood. Here, computational analyses of neutron pair distribution functions of CH_4_, CO_2_ and mixed CH_4_-CO_2_ hydrates at 10 K reveal that the behavior of mixed gas species cannot be interpolated from properties of pure compounds.

## Introduction

Gas hydrates form in natural settings including ocean floor and sub-surface permafrost deposits when water is in the presence of a gas, such as CH_4_, under relatively modest pressures and low temperature conditions. These non-stoichiometric compounds are a hydrogen-bonded water framework which crystallize as a clathrate cage host structure around occluded guest molecules, primarily CH_4_^1–3^. Gas hydrates are high-density sources of CH_4_ and accessible deposits contain an estimated 3000 trillion cubic meters of fuel^2,4^. CH_4_ may be produced from deposits via dissociation (warming or depressurization) or exchange with CO_2_. The latter method can potentially stabilize and maintain natural deposits, while sequestering the hydrocarbon byproduct^4,5^. As thermodynamic predictions, molecular dynamics (MD) simulations, and in situ diffraction experiments demonstrate, a gas hydrate with partial replacement of CO_2_ for CH_4_ forms and is more stable at higher temperature and lower pressure than a pure CH_4_ hydrate^1,3^. This attribute led to a field study in the Alaskan North Slope which demonstrated that CH_4_ can be collected from a hydrate deposit by exchange with CO_2_. The field study, however, did not achieve a complete exchange and resulted in a mixed hydrate deposit^5^. Guest–host interactions of the CH_4_, CO_2_, and mixed CH_4_-CO_2_ hydrate structures need to be thoroughly deciphered, to improve predictions of how to achieve a full CH_4_-CO_2_ exchange and to confirm the stability and safety of the altered deposit with mixed CH_4_-CO_2_ or pure CO_2_ hydrate.

Three clathrate structures have been observed for natural hydrates; however, sI hydrate (the cubic structure type) is the most relevant to the formation conditions of accessible CH_4_ hydrate and therefore our focus in structural studies^1,3,4^. SI hydrate has a cubic $Pm\bar 3n$ (223) crystal structure, composed of 46 hydrogen-bonded H_2_O molecules, which form the host lattice. This structure is made up of eight cages: two small dodecahedral and six large tetrakaidekahedral cages, which can occlude up to one guest molecule^3^. The average crystal structure of sI CH_4_ and CO_2_ hydrate has been well characterized with diffraction^6–11^, spectroscopy^12–15^, MD simulations^16–23^, and density functional theory calculations^11,24–26^, which suggest a high degree of disorder within the crystal. This disorder is evident in the crystallographic model of sI CH_4_ hydrate which requires four partially occupied proton positions for each H_2_O molecule and many positions to represent the unbonded, freely rotating CH_4_ or partially constrained CO_2_ molecules^27^. The intermolecular interactions that result in structural disorder are frequently studied with pair distribution functions (PDFs) calculated from simulated atomic models^18,22,23,28–30^. Computational studies suggest that mixing guests in the hydrate structure impacts the guest–host (CH_4_ or CO_2_–H_2_O), guest–guest, and host–host PDFs. CH_4_ is found to have weaker interactions with the host, providing a more ordered lattice, whereas CO_2_ interacts strongly with the host and other guests, leading to disorder^18,21^. Complementary local structure experiments investigating the impact of CH_4_, CO_2_, and their mixture on the structure and stability of gas hydrates have not been reported to the best of our knowledge. In fact, there are only three published neutron PDF experiments involving gas hydrates which demonstrated limited PDF resolution due to instrumental capabilities^31–33^. Here we present neutron PDF data for CH_4_, CO_2_, and mixed CH_4_–CO_2_ hydrates measured at 10 K using time-of-flight neutron powder diffraction. Detailed analysis of this data was achieved using combined MD and Reverse Monte Carlo (RMC) simulations to model the neutron PDF data. Our analysis provides a path to observe the short-range interactions of CH_4_ and CO_2_ guest molecules with their host hydrate structure and how mixing guest molecules impacts stability.

## Results

### Fitting neutron PDF data

The interpretation of PDFs obtained from neutron scattering experiments of complex materials requires atomic-scale computational modeling. In this work, classical MD simulations of rigid molecules provide an initial structural guess, which we refine with RMC simulation to more accurately explain the PDF data. We approach this data analysis with a combination of MD relaxed models, which are optimized through RMC, which incorporates both physical constraints and experimental observation. Our goal is to provide a descriptive insight into neutron PDF data with features arising from multiple species: in this case, CO_2_, CH_4_, and water.

**Fig. 1:**
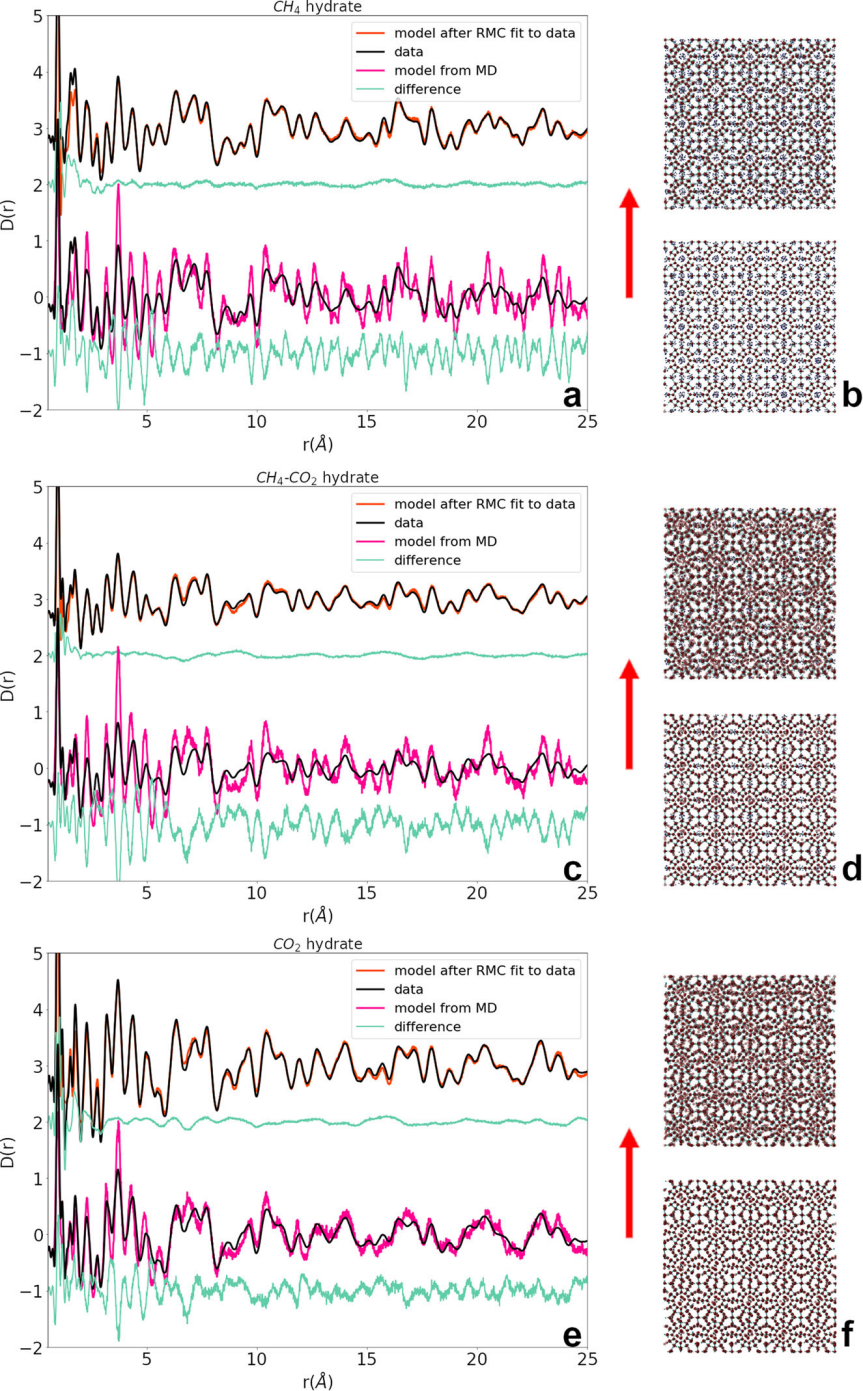
Neutron PDF data and models. Comparison of experimental neutron PDF data with MD modeled PDF (**a**, **c**, **e** bottom spectra) and the model fit to the data with RMC (**a**, **c**, **e** top spectra) with corresponding difference patterns for CH_4_ (**a**), CH_4_–CO_2_ (**c**), and CO_2_ (**e**) hydrates. The overall disorder that RMC models (**b**, **d**, **f** top) calculate is also evident when compared with the MD structures (**b**, **d**, **f** bottom).

Simulated neutron PDFs are calculated from large-box (~20,000 atom) models relaxed with MD, plotted in Fig. 1a, c, e and compared with the experimental neutron PDF data. These model and data comparisons demonstrate the local disorder that is not fully described by the MD simulations of pure CH_4_, pure CO_2_, or mixed CH_4_–CO_2_ hydrate systems, as the simulated peaks are noticeably too sharp and narrow. Although the MD PDFs do predict peak locations and provide a qualitative model of the short-range order in the three hydrate systems, the peak widths and shapes do not match the neutron PDF data. They do not capture the degree of disorder, which represents differences in guest–host interactions across the compositions; however, the MD models provide a good peak location comparison with the neutron data. We implement RMC simulations to further fit this model from MD to the data with RMCProfile. This program is designed for PDF analysis of disordered crystal and amorphous structures, using chemical and physical constraints to fit a large-box model to neutron PDF data^34^. Figure 1a, c, e, demonstrate the improvement achieved when the relaxed MD model for each system is fit to the data with RMC modeling. A dramatic improvement in model to data fit is achieved using RMC for all cases: CH_4_, CO_2_, and mixed CH_4_–CO_2_ hydrates, with a small oscillating difference between the RMC model and the experimental data beyond 2 Å. Pair distances in the 2–10 Å region are the most interesting PDF features for these systems, where CH_4_–CO_2_ composition has the most measurable impact on guest–host and host–host interactions.

The overall lack of disorder in the MD model is the primary source of misfit in Fig. 1 for the atomic separations above 2 Å. We might suspect the rigid intramolecular potentials that were used in the MD simulations as the main culprit, but the improved fits are achieved with RMC constraints which emphasize intermolecular distance relaxations. This is seen, as the greatest RMC-data difference is below 2 Å for all three hydrate systems, where the strictest constraints were applied. We did not pursue further adjustment of the intramolecular bonds to improve this low *r* region or the weak oscillations in the RMC/data difference from 2 to 25 Å, because this risks overfitting and pushes the limitations of the data. Physical constraints are implemented in our RMC simulations to introduce flexible intramolecular bonds, to prevent unphysical collision between atoms and to maintain ice rules for hydrogen bonding in the hydrate host lattice. Under these constraints, the RMC simulation is driven by the goodness of fit of the model to the neutron PDF data^34^.

A comparison of structural snapshots from the MD and RMC simulations visualize the disorder missing in the MD relaxation. The models for all three hydrate compositions, drawn in Fig. 1b, d, f, corresponding to the MD model (bottom) and the RMC fit structure (top), show that the crystal structures are maintained while there is an increase in local disorder of atomic positions. This short-range analysis provides characterization of the local disorder in crystals which is not generally observed in characterization with Bragg diffraction. Achieving PDF analysis of these experimental data enables the investigation of the short-range interactions in these hydrates, which drives varied thermodynamic behavior across the three solid solution compositions.

### Host–host interactions

Once a good fit to the neutron PDF is achieved, we isolate contributions from atom types by calculating radial distribution functions (RDFs) from simulations of the fit models. RDFs are calculated from MD simulations and the RMC fits for analysis of individual species pair distances in the pure CH_4_, CO_2_, and mixed CH_4_–CO_2_ hydrate structures to discern which molecular interactions define the three hydrate systems. RDFs calculated from MD simulation versus RMC fits to experimental data highlight the features that are not captured in the MD simulation. Specifically, host–host, guest–host, and guest–guest intermolecular interactions are compared for the two guest and cage types for both CH_4_ and CO_2_ hydrates, and mixed CH_4_–CO_2_ hydrate via the RDFs calculated from RMC fits. The total neutron PDFs in Fig. 1a, c, e exhibit more disorder in the experimental models than the MD models, so the species-specific RDFs can indicate what interactions lead to that disorder.

**Fig. 2:**
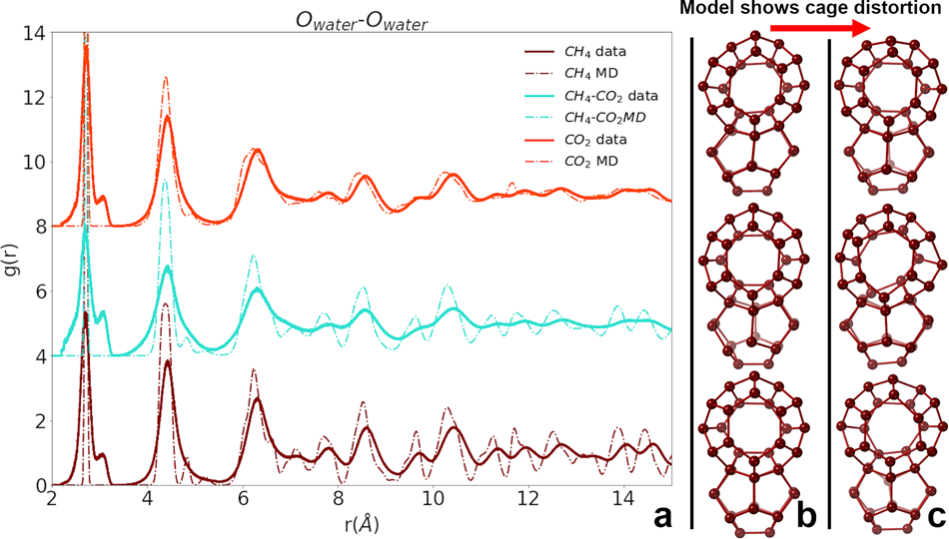
Host–host RDFs. **a** RDFs calculated from MD simulations (dashed lines) and RMC fits to neutron data (solid) for the O_water_–O_water_ pair distances for observing the impact of guest molecule on cage distortion. **b** A large and small cage pair before RMC and **c** after RMC fit to data showing distortion in the cages as observed with neutron PDF data. The distortion of the cages is the most extreme for the mixed guest hydrate (middle row).

Host–host intermolecular interactions are most clearly described by the O_water_–O_water_ RDFs, which are used to measure distortion in the host lattice. RDFs from the fits to neutron data and MD simulations for the pure CH_4_, pure CO_2_, or CH_4_–CO_2_ mixed hydrate samples which compare the impact of guest composition on the hydrate host lattice are shown in Fig. 2a. Structures of large and small cages selected from the large-box models are shown in Fig. 2b, c to visualize the MD and the experimental results. The RDF peaks in Fig. 2a, which measure O_water_–O_water_ pair distances for the D_2_O molecules that form hydrate cages, are more distorted in the experiment than MD models predict and are generally wider, shorter, and not normally distributed. Analysis of the experimental data shows that the first nearest neighbor O_water_–O_water_ peak, at ~2.8 Å, is much broader than MD simulations indicate. Instead of normally, tightly distributed distances, the O pairs distribute from 2 to 3.25 Å with an average of 2.7 Å and a defined high-*r* shoulder at 3.2 Å. The mixed CH_4_–CO_2_ hydrate also has a low *r* shoulder due to some shortening of pentagonal face edges. This detail, in combination with the smeared peak shape as *r* increases, indicates that the hydrate lattice is the most disordered for a guest composition of mixed CH_4_–CO_2_. We can visualize a large and small cage extracted from experimental fits in Fig. 2c to show that mixed CH_4_–CO_2_ hydrate cages are the most distorted. As these compositions follow experimental occupancy, some CH_4_ and the mixed CH_4_–CO_2_ hydrate cages are vacant. The cages drawn in Fig. 2b, c were chosen as ones where the large cage is occupied by a CO_2_ and the small cage is occupied by a CH_4_. The imbalance of guest molecule shape and interaction potential in the mixed hydrate system, which arises from the cage filling where CO_2_ may be surrounded by CH_4_ or a vacancy, clearly leads to higher level of disorder in the structure.

### Guest–host Interactions

**Fig. 3:**
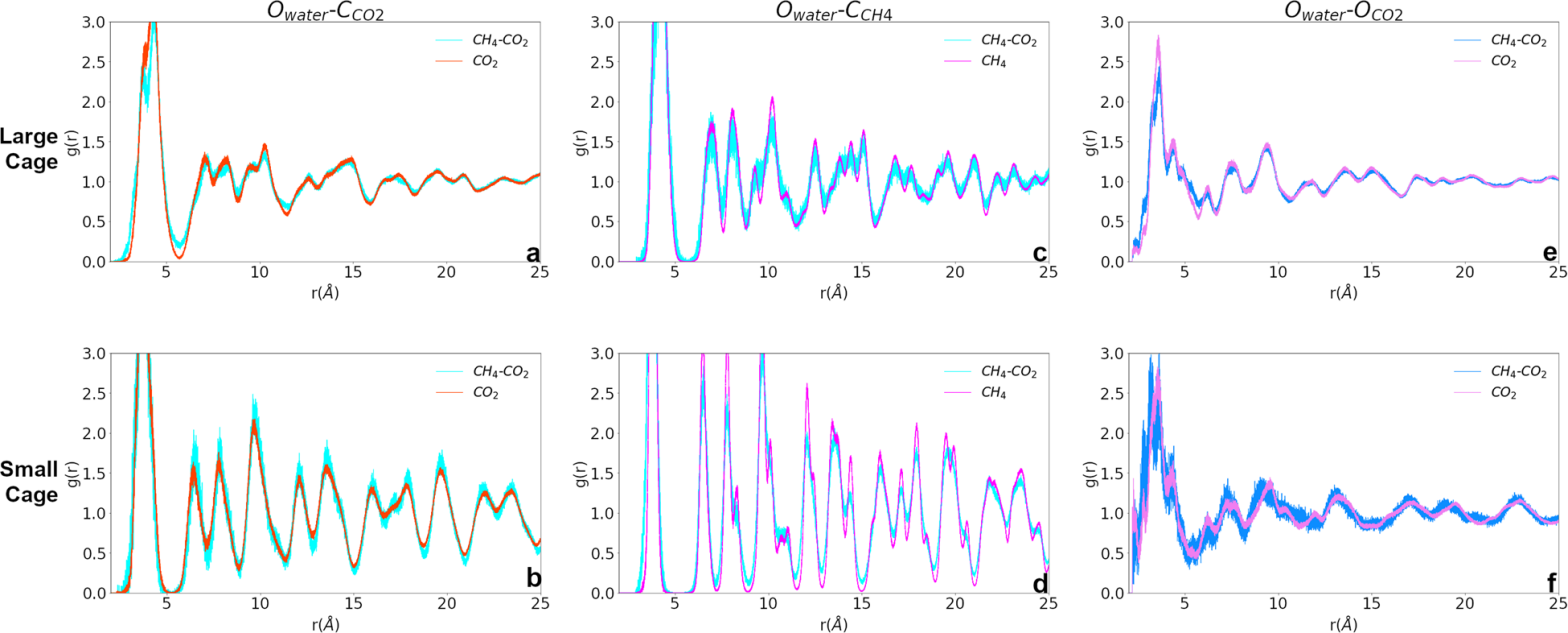
Host–guest RDFs. RDFs to investigate guest–host interactions in three hydrate compositions. O_water_–C_CO2_ RDFs for the large cage (**a**) and small cage (**b**) CO_2_ centers and O_water_–C_CH4_ RDFs for the large cage (**c**) and small cage (**d**) CH_4_ centers indicate that CO_2_ molecules are not as ordered in their positions as CH_4_. Orientations of the linear CO_2_ in O_water_–O_CO2_ RDFs in the large cage (**e**) and small cage (**f**) show that the CO_2_ in mixed hydrate orients in more positions towards the cage.

A range of cage filling results have been predicted and observed for hydrates synthesized with CH_4_ or CO_2_ as the feed gas under varying pressure–temperature conditions and synthesis procedures. At 10 K, despite the larger size of the CO_2_ molecule, hydrate with CO_2_ as a guest has a smaller lattice parameter and CO_2_ will preferentially fill the large cage over CH_4_^3,7^. Local structure analysis of the guest–host RDFs in Fig. 3 lead to an understanding of how the guest molecules in different compositions interact with the host and affect cage filling, formation, stability, and decomposition behavior^3,7,18^. RDFs corresponding to the CO_2_ molecule center (the central C) for pure CO_2_ and mixed CH_4_–CO_2_ hydrates are plotted in the top row of Fig. 3 for the large cage (a) and small cage (b). The first peak for the O_water_–C_CO2_ pair distances is centered around ~4.5 Å for both compositions, but the mixed hydrate has a distinct split and is wider. The CO_2_ guest in the mixed hydrate is located at more distinct positions than in the pure CO_2_ hydrate, where the molecule is primarily centrally located in the cage. Overall, though, the guest RDF peaks in the large cage show that the local guest–host structure of CO_2_ in mixed hydrate is more disordered. This will impact stability upon heating and suggests that host–CO_2_ interactions in mixed hydrate are more favorable than in pure CO_2_ hydrate. In the small cage (Fig. 3b), the position of the molecule center is essentially the same in both compositions. The CH_4_ molecule in CH_4_ and mixed hydrate systems occupies a tighter distribution of orientations than CO_2_. The O_water_–C_CH4_ RDFs in the middle row (Fig. 3c, d) are defined by sharper, narrower peaks than in the CO_2_ hydrate cases. Though the CH_4_ positions are slightly less ordered in the mixed hydrate composition, the CH_4_ guest has a lower interaction potential with the other molecules in the system, is not a polar molecule, and essentially occupies cage centers. The quadrupolar CO_2_ occupies a more distributed position, especially in the large cage, leading to different interaction surfaces with its immediate host lattice D_2_O neighbors. Finally, as CO_2_ is a linear molecule, the O_water_–O_CO2_ RDFs in the bottom row of Fig. 3e, f show that CO_2_ in both cages approaches the D_2_O molecules at distances just above 2 Å, close enough to form weak hydrogen bonds which result in the distortions observed in Fig. 2 and structural stabilization. Guest–host distances across the RDFs of both compositions show that largest distortion of the local hydrate cage structure occurs in the mixed hydrate system. In this composition, CO_2_ may be neighbored by another CO_2_, CH_4_, or a vacancy. We see that a change in surrounding environment allows the molecule to distribute across a broader range of positions and orientations in the cages, causing O_CO2_ to interact more closely with enclathrating D_2_O molecules.

**Fig. 4:**
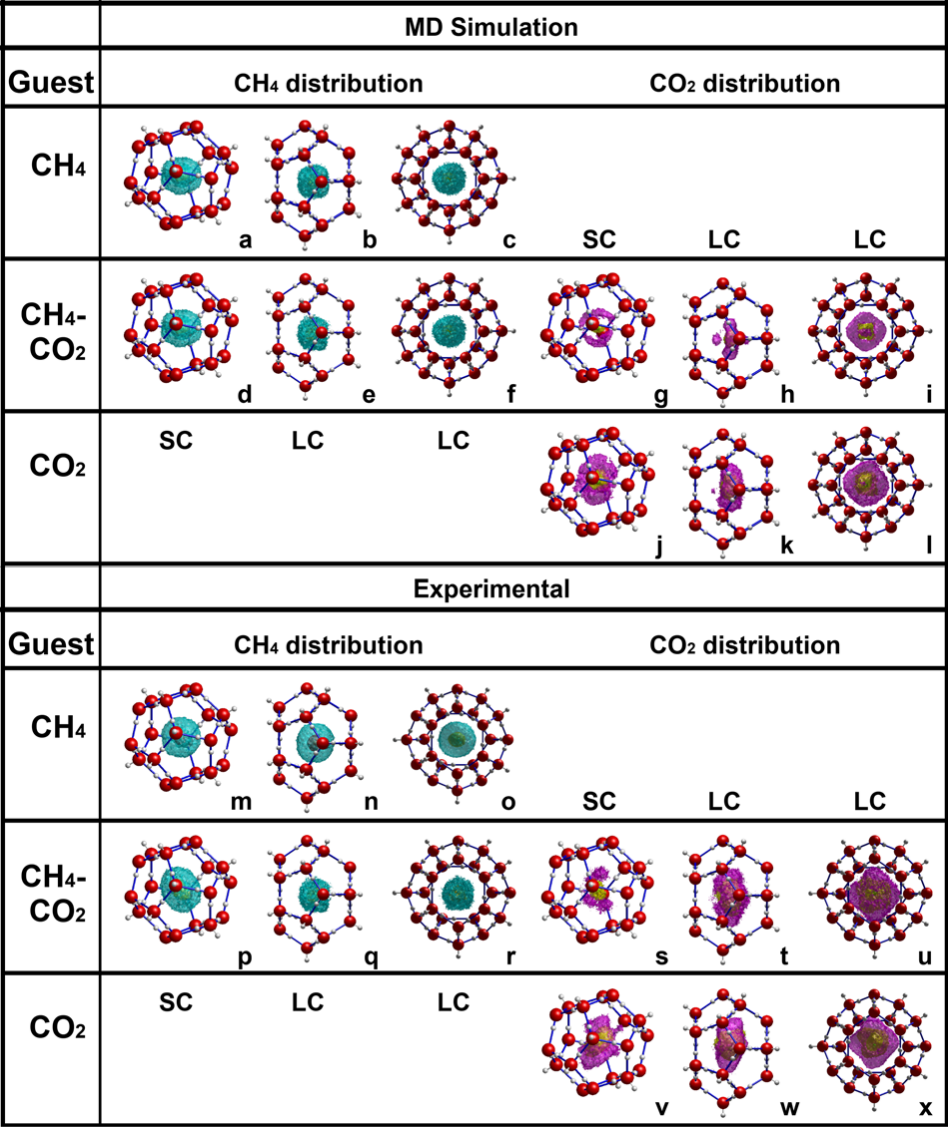
Distribution of CH_4_ and CO_2_ in cages. Three-dimensional distributions, calculated from the trajectory frames from MD simulations (**a**–**l**) and from the sampled frames from the RMC fits (**m**–**x**) that were used to make the RDFs.

We can visualize and better understand the RDFs with three-dimensional density distributions calculated from MD trajectory frames and RMC fits. Orientation rotations were performed on the large cage guest molecules in order to accurately describe how they occupy the cage space with respect to the two offset hexagonal faces. Consistent with former experimental and computational results, the CH_4_ molecule in both the MD and experimental distributions in Fig. 4a–f, m–r, respectively, orients with a spherical distribution in both the large and small cages^7,18^. Previously, the CO_2_ molecule was shown to orient in an oblate shape parallel to the hexagonal face of the large hydrate cage, along the longer axis^7,10,24,35^. Neutron diffraction experiments showed this orientation in both pure CO_2_ and mixed CH_4_–CO_2_ hydrate, but MD simulations showed that this only held for distributions in the pure CO_2_ hydrate. MD simulations in Fig. 4h, i show CO_2_ occupying an additional orientation towards the hexagonal face across the short axis of the cage, elaborating on previous diffraction studies where this orientation seems to be averaged out in the long-range crystallographic analysis. Neutron PDF analysis allows for direct experimental evidence of this local structure for the mixed hydrate. Figure 4t, u, w, x provide density distributions of the CO_2_ produced from models of the experimental data, which indicate that in mixed CH_4_–CO_2_ hydrate the guest in the large cage does occupy additional orientations toward the hexagonal face that do not occur in CO_2_ hydrate. Experimental orientations are not as defined as MD predicts, as might be expected from the trend of higher disorder in experimental data seen throughout the model comparisons in previous sections. We do see, however, a low density of orientations pointing directly into the hexagonal face and greater distribution of O_CO2_ positions angling toward that face than in the pure CO_2_ composition where a narrower oblate distribution is occupied.

## Discussion

Guest–host interactions in CH_4_, CO_2_, and mixed CH_4_–CO_2_ hydrates, as observed by experimental neutron PDF analysis, are dependent on guest type and cage filling. The mixed hydrate local structure is more disordered and distorted than the pure CH_4_ and CO_2_ hydrates. Although these solid solution compositions follow a rule of mixtures for crystallographic properties including lattice parameter, the mixed hydrate is the outlier when the local structure is inspected. Large-box models were fit to neutron PDF data for CH_4_, CO_2_, and mixed CH_4_–CO_2_ hydrate powders, to produce RDFs and density distributions for local structure analysis. CO_2_, when surrounded by CH_4_ and vacant cages, has the freedom to move into a broader distribution of orientations in the hydrate cages. This is an important detail to consider when attempting a CH_4_–CO_2_ exchange, as a mixed hydrate will form in the intermediate steps. RDFs show that the local structure of the occluded species in the mixed hydrate clearly does not fall between that in the pure CH_4_ and CO_2_ hydrates. Guest molecule interactions with the host lattice cannot be expected to behave as an intermediate of pure CH_4_ and CO_2_ hydrate, even though predicted pressure and temperature of formation from gas and liquid components do. The altered CO_2_–host local structure and interactions may create a free energy well that requires an interruption or altered temperature/pressure input to overcome as the disordered positions are more closely interacting with the host molecules. Our analysis approach provides a path forward to analyze neutron PDF experiments of an in situ CH_4_–CO_2_ exchange. Developing these studies allows for investigation into possible remedies to achieve a full CH_4_–CO_2_ exchange, such as a helper molecule to overcome the energy barrier^5^. Characterization of guest–host structure and interactions leads to understanding of the long-term stability of an altered natural hydrate deposit. This local characterization at 10 K shows that framework distortion in mixed CH_4_–CO_2_ hydrate, stabilized by CO_2_, results in a disordered structure that inhibits further CH_4_ removal. We demonstrate that the CH_4_–CO_2_ guest composition impacts the guest–host interactions in hydrates during static temperature measurements at 10 K, implying that these interactions are important in formation and decomposition. Further studies should apply these techniques during those reactions if a complete structure–property relationship is to be determined. These experimental and analysis methods have successfully presented observable differences in the hydrate compositions with CH_4_ and CO_2_ at 10 K. The development of these experiments and analysis at cold temperatures, where the complexity of molecular thermal motion of is minimal, makes investigations such as this possible at the higher working temperatures of a CH_4_–CO_2_ exchange.

## Methods

### Sample synthesis

CH_4_, CO_2_, and mixed CH_4_–CO_2_ hydrate powders were synthesized for the neutron total scattering experiments. One milligram of *Pseudomonas syringae 31a*, branded as Snomax, was mixed with 10 mL D_2_O for each sample. Snomax is an ice nucleating protein and has been demonstrated to decrease the formation pressure required for gas hydrates^35^. A 300 mL pressure reactor vessel containing the solution and steel milling media was evacuated and then pressurized at room temperature with 600 psi of gas. The three samples were pure CO_2_ and CH_4_ hydrates, and mixed CH_4_–CO_2_ hydrates, where the CH_4_/CO_2_ feed gas fraction was 0.5/0.5. For each composition after pressurization, the vessel was placed in a freezer where the temperature was dropped to 277 K and tumbled periodically for ~7 days. The freezer temperature was dropped to 253 K for at least 1 day to quench the sample after the pressure in the vessel equilibrated. Hydrate powders were collected under a nitrogen atmosphere and stored in vanadium cans in liquid nitrogen until the neutron experiments.

### Measuring neutron PDFs

Neutron PDF data were collected from CH_4_, CO_2_, and mixed CH_4_–CO_2_ hydrate powder samples on the Nanaoscale-Ordered Materials Diffractometer at the Spallation Neutron Source at Oak Ridge National Laboratory. Samples were measured at 10 K in a 50 mm He cryostat. Vanadium cans containing each sample were transferred from the liquid nitrogen storage dry shipper to the cryostat set to 90 K. The sample was then held in the cryostat at 90 K, to allow any liquid nitrogen to boil off before temperature was dropped to 2 K and increased to 10 K. Data were collected under low He pressure at 55 mbar, the working pressure for the cryostat. Neutron PDF data were collected in reciprocal space and Fourier transformed to obtain the real space function. The neutron *S(Q)s* from 0.6-35 Å^−1^ were converted in PyStoG^36^ to the real space function using

1 $$G\left( r \right) = \frac{2}{\pi }\int_{Q_{\mathrm{min}}}^{Q_{\mathrm{max}}} {F\left( Q \right){\mathrm{sin}}\left( {Qr} \right)dQ = 4\pi rp_0\left( {g\left( r \right) - 1} \right)}$$

with

2 $$F\left( Q \right) = Q\left( {S\left( Q \right) - 1} \right)$$

and the intermolecular bond distances (greater than ~2.5 Å) are emphasized by weighting the function as

3 $$D\left( r \right) = 4\pi r\rho G\left( r \right)$$

which is the function that was used to fit all neutron PDF data^37,38^.

Measuring a neutron PDF is achieved by collecting data through a wide range of *Q* to observe Bragg reflections and diffuse scattering, providing a powder diffraction pattern for crystal structure analysis. Rietveld refinements of the Bragg data to confirm the hydrate structure, obtain average composition and lattice parameters, and amount of ice (present as a minor secondary phase) were performed using GSAS-EXPGUI^39,40^. The details are outlined in Supplementary Table 1 and Supplementary Fig. 1, and are in agreement with the results for the same three compositions synthesized under the same conditions measured with high-resolution neutron powder diffraction at 10 K^7^.

### MD simulations and PDF analysis

Large-box models of ~20,000 atoms (depending on composition) were produced by building 5 × 5 × 5 unit cell models of the host lattice following the ideal proton configuration for sI hydrates determined by Takeuchi et al.^27^. The large and small cages were filled with CH_4_, CO_2_, or a vacancy according to the experimentally determined occupancies, outlined in Supplementary Table 2^7^. Two sets of models were built with this method for the three hydrate compositions using experimental lattice parameters. One set of initial large boxes were equilibrated with the LAMMPS (large-scale atomic/molecular massively parallel simulator) simulations package in the NPT (constant atoms, pressure, and temperature) and then simulated in the NVT (constant atoms, volume, and temperature) ensemble for 100 ps with a 1 fs timestep and 100 fs damping constant^41^. RDFs and density distributions were calculated from the NVT trajectories to compare with the experimental results. Fixed rigid models were used in the MD simulations for the H_2_O, CH_4_, and CO_2_ molecules. The TIP4P potential model with a long-range Coulombic solver was used for the water molecules, while the CH_4_ and CO_2_ partial charges and Lennard Jones parameters were taken from Tse et al.^30^ (CH_4_) and Duan and Zhang^42^ (CO_2_). Finally, all intermolecular interactions for H_2_O, CH_4_, and CO_2_ were determined with Lorentz–Berthelot combination rules^22^. The second set of boxes were relaxed in NVT for 100 ps at the lattice parameter and composition determined with Rietveld refinements to produce initial models for RMC fits.

Large-box models were fit to the data using RMCProfile^34^. RMC model constraints, summarized in Supplementary Table 3, result in an acceptance rate of ~10% once equilibration is reached. RDFs and density distributions were calculated from the equilibrated MD trajectories and sets of frames from RMC fits using custom codes written in FORTRAN. RMC fits for the CH_4_, CO_2_, and mixed CH_4_–CO_2_ hydrate models were each simulated for an additional 25 × 10^6^ generated moves from 15 unique initial configurations and the resulting structures were sampled to produce a collection of ~200 frames of accepted atom configurations for each structure. We mimicked MD simulation trajectories from equilibrated RMC frames and calculated partial RDFs for individual species pair distances in the three structures. These RDFs are a probability density function, which describe the likelihood of a particle being at a distance *r* from another particle and are calculated as:

4 $$\int_0^\infty {\rho g\left( r \right)4\pi r^2dr = N \approx N - 1}$$

where $g\left( r \right)$has the limits as $r \to \infty$, $g \to 1$^37^.

## Supplementary information

Supplementary Information

Peer Review File

## Data Availability

The data that support the findings of this study are available from the corresponding author upon request.

## Code availability

The codes used in this study are primarily cited. RMCProfile version 6.7.4.3 was used to fit the neutron data and is available at RMCProfile.org. PyStog version 0.1.3 is available at https://pystog.readthedocs.io/en/latest/. The code to calculate the radial distribution function from data fits and simulations is found at http://utkstair.org/clausius/docs/mse614/text/examples.html. MD simulations were performed with LAMMPS, documented at https://lammps.sandia.gov/doc/Manual.html.
