# Supplementary material for: Local structure and distortions of mixed methane-carbon dioxide hydrates
Source: Commun Chem. 2021 Jan 19;4:6. doi: 10.1038/s42004-020-00441-7 (PMC9814247; doi:10.1038/s42004-020-00441-7)
Supplement: Supplementary file 1 — Supplementary Information [file 42004_2020_441_MOESM1_ESM.pdf]

Supplementary Information

Supplementary Table 1. Lattice parameters from the Rietveld refinements and hydrate/ice phase fractions from the Bragg patters obtained from the total scattering instrument (NOMAD) and from high resolution neutron powder diffraction (POWGEN) in previous work.

Feed gas composition	Lattice parameter (Å)		Phase fraction ice
	NOMAD (this work)	POWGEN (Everett et al.[1])	NOMAD (this work)
**100% CH_4**	11.830(2)	11.83210(8)	0.10(1)
**50% CH_4 50% CO_2**	11.8244(3)	11.82487(8)	0.21(1)
**100% CO_2**	11.8192(7)	11.82216(9)	0.13(1)

Supplementary Table 2. Hydrate cage occupancies high resolution neutron powder diffraction (POWGEN) in previous work.[1]

Feed gas composition	Large cage occupancy		Small cage occupancy		Total composition %		Total cages filled %
	CH_4	CO_2	CH_4	CO_2	CH_4	CO_2	
**100% CH_4**	0.73(3)	-	0.93(5)	-	79(4)	-	79(4)
**50% CH_4 50% CO_2**	0.08(3)	0.77(3)	0.54(4)	0.21(2)	20(3)	63(1)	83(5)
**100% CO_2**	-	1.00(6)	-	1.00(4)	-	100(6)	100(6)

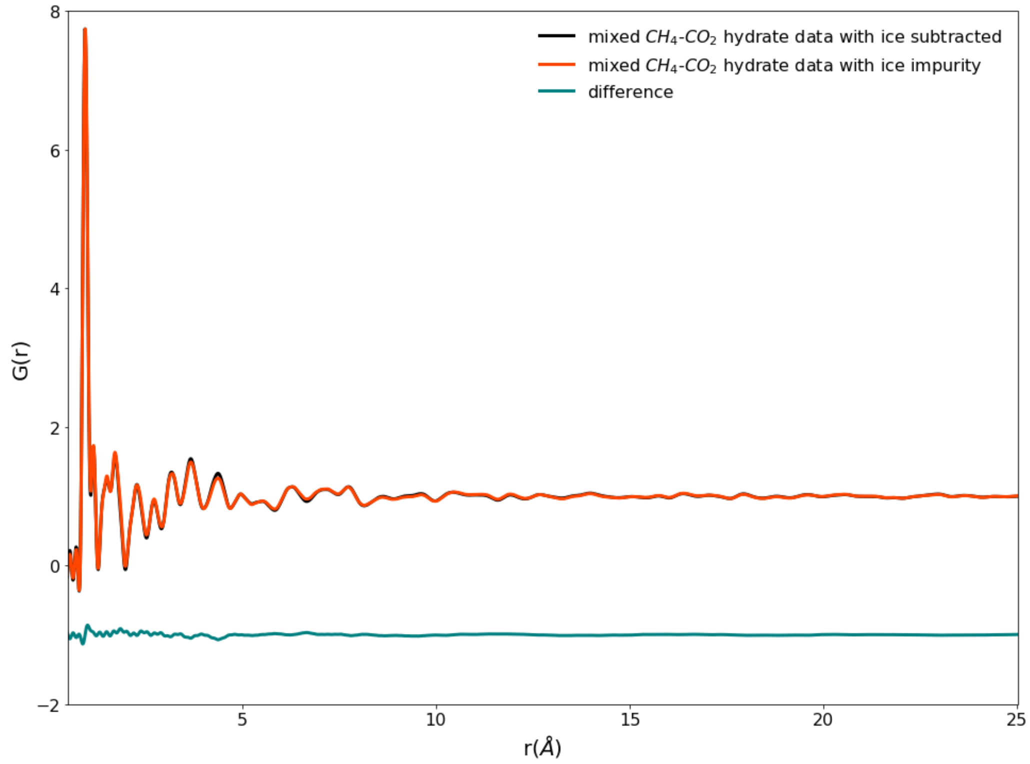

Supplementary Figure 1. Comparison of mixed CH_4-CO_2 hydrate PDF data with the ice impurity and with ice subtracted shows that the impurity has little effect on the PDF.

Supplementary Table 3. Summary of physical constraints applied in the RMC simulations.

**Closest approach pair distances (Å)**									
O-O	O-D	O-C	O-H	D-D	D-C	D-H	C-C	C-H	H-H
2.2	0.85	1.1	2.5	1.2	2.5	2.0	5.0	1.0	1.5

**Maximum distance per move (Å)**			
O	D	C	H
0.05	0.05	0.05	0.05

**Distance window for O-D hydrogen bonded pairs (Å)**	
Minimum distance	Maximum distance
1.6	2.25

Supplementary References

1 Everett, S. M. *et al.* Insights into the structure of mixed CO_2/CH_4 in gas hydrates. *American Mineralogist* **100**, 1203-1028 (2015).
